# AT-hook peptides bind the major and minor groove of AT-rich DNA duplexes

**DOI:** 10.1093/nar/gkac115

**Published:** 2022-02-25

**Authors:** Alyssa Garabedian, Kevin Jeanne Dit Fouque, Prem P Chapagain, Fenfei Leng, Francisco Fernandez-Lima

**Affiliations:** Department of Chemistry and Biochemistry, Florida International University, Miami, 33199, USA; Department of Chemistry and Biochemistry, Florida International University, Miami, 33199, USA; Biomolecular Sciences Institute, Florida International University, Miami, 33199, USA; Department of Physics, Florida International University, Miami, 33199, USA; Biomolecular Sciences Institute, Florida International University, Miami, 33199, USA; Department of Chemistry and Biochemistry, Florida International University, Miami, 33199, USA; Biomolecular Sciences Institute, Florida International University, Miami, 33199, USA; Department of Chemistry and Biochemistry, Florida International University, Miami, 33199, USA; Biomolecular Sciences Institute, Florida International University, Miami, 33199, USA

## Abstract

The mammalian high mobility group protein AT-hook 2 (HMGA2) houses three motifs that preferentially bind short stretches of AT-rich DNA regions. These DNA binding motifs, known as ‘AT-hooks’, are traditionally characterized as being unstructured. Upon binding to AT-rich DNA, they form ordered assemblies. It is this disordered-to-ordered transition that has implicated HMGA2 as a protein actively involved in many biological processes, with abnormal HMGA expression linked to a variety of health problems including diabetes, obesity, and oncogenesis. In the current work, the solution binding dynamics of the three ‘AT-hook’ peptides (ATHPs) with AT-rich DNA hairpin substrates were studied using DNA UV melting studies, fluorescence spectroscopy, native ion mobility spectrometry-mass spectrometry (IMS-MS), solution isothermal titration calorimetry (ITC) and molecular modeling. Results showed that the ATHPs bind to the DNA to form a single, 1:1 and 2:1, ‘key-locked’ conformational ensemble. The molecular models showed that 1:1 and 2:1 complex formation is driven by the capacity of the ATHPs to bind to the minor and major grooves of the AT-rich DNA oligomers. Complementary solution ITC results confirmed that the 2:1 stoichiometry of ATHP: DNA is originated under native conditions in solution.

## Graphical Abstract

**Graphical Abstract:**
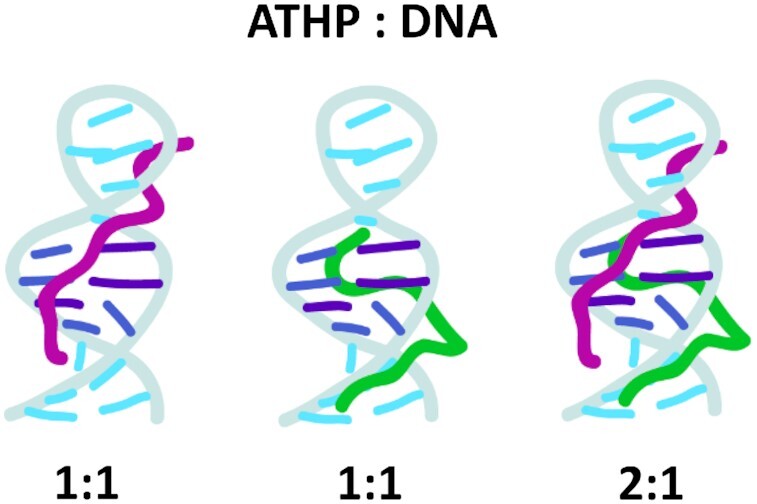
AT-hook peptides 1, 2 and 3 from HMGA 2 bind to the major and minor groove of AT-rich DNA duplexes to form a 1:1 and 2:1 ‘key-locked’ conformational ensemble.

## INTRODUCTION

The mammalian high mobility group protein AT-hook 2 (HMGA2) is a small non-histone chromosomal protein and belongs to the HMGA protein family (1,2). It has three ‘AT-hook’ DNA binding motifs and specifically binds to AT-rich DNA sequences (3–6). The ‘AT-hook’ DNA-binding motif is an 8–9 amino acid peptide with a conserved tripeptide -Arg-Gly-Arg- core surrounded by multiple positively charged amino acids lysines and/or arginines (3). HMGA2 is a multifunctional nuclear protein and is likely a key player for several important biological processes that yield certain unique phenotypes. For instance, HMGA2 is linked to obesity (7,8), diabetes (9), stem cell youth (10), and oncogenesis (11–14). HMGA2 is also associated with human height (15–17) and intelligence (18). Due to the importance of HMGA2 in adipogenesis (19) and tumorigenesis (20), HMGA2 is considered a potential therapeutic target for anticancer and anti-obesity treatments (8,21–23).

HMGA2 is an intrinsically disordered protein (IDP) (24,25) and does not have a secondary and tertiary structure in the absence of DNA. When the ‘AT-hook’ DNA bind motifs bind to AT-rich DNA sequences, they adopt a defined structure (24,26). Specifically, the central RGR core deeply penetrates into the minor groove of AT base pairs and forms a well-defined ‘AT-hook’ and DNA complex (24,26). Interestingly, recent mass spectrometry and simulation studies showed that certain structures of the ‘AT-hooks’ are more stable and similar to the structures observed in the NMR and crystal structural studies (2,11), suggesting that the role of AT-rich DNA is to stabilize these structures or conformations.

Previously, using a PCR-based systematic evolution of ligands by exponential enrichment (SELEX) experiment, we found that HMGA2 prefers binding to the following two consensus DNA sequences, 5′-ATATTCGCGAWWATT-3′, and 5′-ATATTGCGCAWWATT-3′, where W represents A or T (5). Intriguingly, these two HMGA2 binding sequences contain a 4-base pair GC sequence in the center. Because each ‘AT-hook’ binds to 5 base pairs and the minor groove of the GC sequence is crowded, one ‘AT-hook’ likely interacts primarily with the major groove of the center GC sequence. This hypothesis prompted us to investigate whether the ‘AT-hooks’ also tightly bind to the DNA major groove if the minor groove is not available. Since a variety of DNA-binding proteins carry ‘AT-hook’ DNA-binding motifs (27), not only will the discovery/confirmation of ‘AT-hooks’ binding to the DNA major groove change our view toward this DNA binding motif, but it also has great biological consequences. One implication is that HMGA proteins or DNA-binding protein carrying ‘AT-hook’ motifs, if minor groove is not available, can still interact with DNA through major groove and participate in nuclear activities, such as transcription, chromatin remolding, and DNA repair.

One approach to address this hypothesis is to study how the ‘AT-hook’ peptides (ATHPs) derived from the three ‘AT-hook’ DNA binding motifs of HMGA2 bind to DNA molecules containing only one AT-rich DNA binding site. In the present work, we report on the binding dynamics of the three ATHPs of HMGA2 with AT-rich DNA hairpin substrates using DNA UV melting studies, florescence spectroscopy, isothermal titration calorimetry (ITC), ion mobility spectrometry – mass spectrometry (IMS-MS) and molecular modeling. Although prior studies suggested minor groove ATHP interactions, here we present, for the first time, solution- and gas-phase experimental and theoretical evidence of both ATHP major and minor DNA groove-binding capabilities.

## MATERIALS AND METHODS

### Materials and reagents

**Scheme 1.**
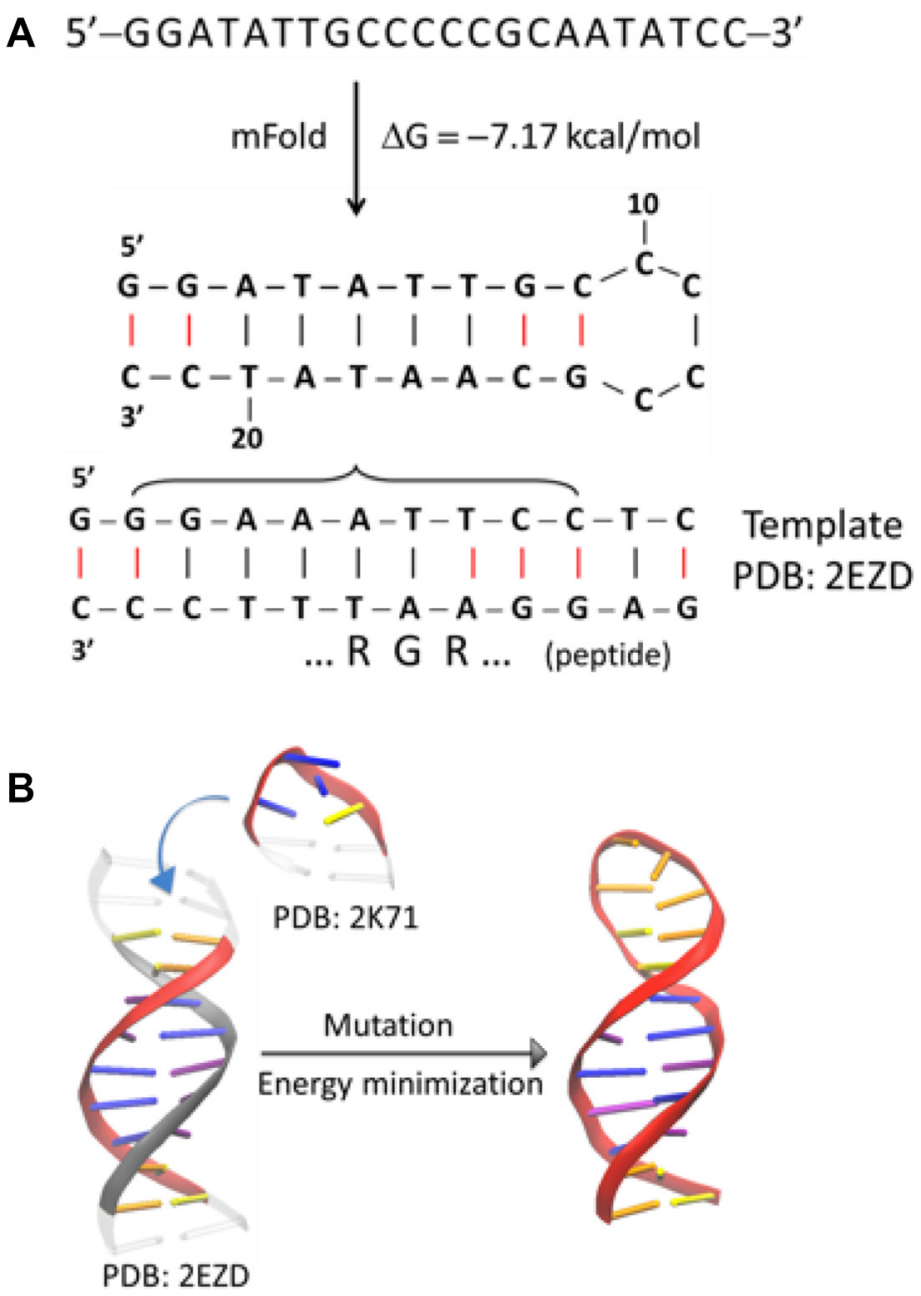
Preparation of PDB template for modeling of DNA.

AT-rich DNA oligomers, FL876 (sequence: 5′-GGATATTGCCCCCGCAATATCC-3′ (C_212_H_270_N_79_O_130_P_21_, MW 6655.1561)), FL876T1 (sequence: 5′-GGATATTGCCTCCGCAATATCC-3′ (C_213_H_272_N_78_O_131_P_21_, MW 6670.3326), and FL876T2 (sequence: 5′-GGATATTGCCTTCGCAATATCC-3′ (C_214_H_273_N_77_O_132_P_21_, MW6685.3440)) were purchased from Eurofins Genomics (Luxembourg, Luxembourg) and used as received (Scheme 1 and Supplementary Scheme S1). These three 22 nucleotide DNA hairpins contain a 9-base pair stem including a 5 base pair AT DNA in the middle of the stem. AT-hook peptides 1 (Lys-Arg-Gly-Arg-Gly-Arg-Pro-Arg-Lys), 2 (Pro-Lys-Arg-Pro-Arg-Gly-Arg-Pro-Lys) and 3 (Lys-Arg-Pro-Arg-Gly-Arg-Pro-Arg-Lys-Trp), which correspond to the first, second, and third ‘AT-hook’ motifs of HMGA2, were purchased from Advanced ChemTech Inc. (Louisville, KY) and used as received. Solvents, 1xBPE buffer (6 mM Na_2_HPO_4_, 2 mM NaH_2_PO_4_, and 1 mM Na_2_EDTA, pH 7.0), and ammonium acetate salts utilized in this study were analytical grade or better and purchased from Fisher Scientific (Pittsburgh, PA). A Tuning Mix calibration standard (G24221A) was obtained from Agilent Technologies (Santa Clara, CA) and used as received.

### DNA UV melting studies

DNA UV melting curves were determined using a Cary 100 UV-Vis spectrophotometer (Agilent Technologies, Santa Clara, CA) equipped with a thermoelectric temperature-controller. DNA oligomer FL876 in 1 × BPE buffer (16 mM of Na^+^) was used for melting studies. Under this condition, the AT-rich DNA site of FL876 is in the double-stranded form at room temperature. DNA-peptide samples were prepared to a final concentration of 2 μM by directly mixing at a molar ratio of 1:10 FL876 DNA with each ATHP peptide, followed by incubation for 60 min at room temperature to ensure equilibration. Samples were typically heated in the 20–100°C range at a rate of 1°C min^–1^, while continuously monitoring the absorbance at 260 nm. Primary data were transferred to the graphic program Origin (MicroCal, Inc., Northampton, MA) for plotting and analysis.

### Fluorescence measurements

Fluorescence spectra of DNA-ATHPs were acquired using a Jobin Yvin Horiba FluoroMax-3 with excitation wavelength of 355 nm. In the titration experiments, 99 nM FL876 and 50 nM Hoechst stain in 1 × BPE was titrated by increasing concentrations of ATHP 1, 2, and 3 up to 20 μM. The fluorescence spectra were recorded from 400 to 550 nm.

### Isothermal titration calorimetry (ITC)

ITC experiments were performed using a VP-ITC titration calorimeter (MicroCal, Inc., Northampton, MA) interfaced to a personal computer. Origin 7.0 was used for data acquisition and processing. A typical ITC experiment was set up so that 10 μL of ATHP ligand, i.e., 300 μM ATHP1, 300 μM ATHP2 and 150 μM ATHP3, was injected every 120 seconds for 30 injections into a 10 μM DNA sample (1xBPE buffer) in the sample cell.

### Native trapped ion mobility spectrometry – mass spectrometry analysis (TIMS-MS)

Details regarding the TIMS operation and specifics compared to traditional IMS can be found elsewhere (11,28–31). Briefly, mobility separation in TIMS is based on holding the ions stationary against a moving gas using an electric field. The separation in a TIMS device can be described in the center of the mass reference frame using the same principles as in a conventional IMS drift tube (32). Since mobility separation is related to the number of ion-neutral collisions (or drift time in traditional drift tube cells), the mobility separation in a TIMS device depends on the bath gas drift velocity, ion confinement and ion elution parameters. The reduced mobility, *K*, of an ion in a TIMS cell is described by:

$$\begin{equation*}K\ = {\rm{\ \ }}\frac{{{V_g}}}{E} = {\rm{\ }}\frac{A}{{\left( {{V_{elution}} - {V_{out}}} \right)}}\end{equation*}$$

where v_g_ and E are the velocity of the gas and the applied electric field across the TIMS analyzer region. V_elution_ is the voltage when the ions elute in the V_ramp_ sweep and V_out_ is the voltage applied at the end of the TIMS analyzer region.

A custom-built, pulled capillary nanoESI source was utilized for all the experiments. Quartz glass capillaries (O.D.: 1.0 mm and I.D.: 0.70 mm) were pulled utilizing a P-2000 micropipette laser puller (Sutter Instruments, Novato, CA) and loaded with 10 μL aliquot of the sample solution. Sample solutions consisted of 1–10 μM ATHP or DNA in 10 mM ammonium acetate solution at physiological pH (pH = 6.7). For the observation of the DNA-ATHP complexes, a 1:1 and 1:2 ratio of 5 μM concentration of the DNA and AT-hook peptide (1, 2 or 3) was prepared in 10mM ammonium acetate immediately prior infusion. A typical nanoESI source voltage of +/- 600–1200 V was applied between the pulled capillary tips and the TIMS-MS instrument inlet. Ions were introduced via a stainless-steel tube (1/16 × 0.020′, IDEX Health Science, Oak Harbor, WA) held at room temperature into the TIMS cell.

Mobility calibration was performed using the Tuning Mix calibration standard (G24221A, Agilent Technologies, Santa Clara, CA) in positive ion mode (e.g., *m/z* = 322, K_0_ = 1.376 cm^2^ V^–1^ s^–1^ and *m/z* = 622, K_0_ = 1.013 cm^2^ V^–1^ s^–1^) (31). The TIMS operation was controlled using in-house software, written in National Instruments Lab VIEW, and synchronized with the maXis Impact Q-ToF acquisition program (28,29).

Reduced mobility values (K_0_) were correlated with CCS (Ω) using the equation:

$$\begin{equation*}{\rm{\Omega \ }} = \frac{{{{\left( {18\pi } \right)}^{1/2}}}}{{16}}\ \frac{z}{{{{({k_B}T)}^{1/2}}}}{\left[\frac{1}{{{m_i}}} + \frac{1}{{{m_b}}}\right]^{1/2}}\frac{1}{{{K_0}}}\frac{1}{{{N^{\rm{*}}}}}\end{equation*}$$

where z is the charge of the ion, kB is the Boltzmann constant, N* is the number density and mI and mb refer to the masses of the ion and bath gas, respectively (32).

### Molecular modeling

Candidate structures were proposed for the mobility bands observed in the TIMS-MS experiments. The protein data bank (PDB) entry 2EZD was used as a template for the peptide-DNA candidate structures. First, a 2-dimensional DNA hairpin structure was obtained for the DNA sequence using mFold server (33). Based on this structure, a 3-dimensional hairpin structure was then created by merging PDBs 2EZD (for the stem) with 2K71 (for the tetra-nucleotide loop) followed by mutations with appropriate bases to achieve the desired structure (see Scheme 1). The modeled DNA hairpin structure was optimized by energy minimization with CHARMM36 force field (34) using NAMD Molecular Dynamics package (35). Molecular docking was performed using AutoDock vina (36) to generate the DNA-ATHP complexes. Theoretical CCS were calculated using the IMoS (v1.04b) (37–39) and PSA (40) packages with nitrogen as a bath gas at ca. 300K. In the IMoS calculations, 100 total rotations were performed using the trajectory method with a Maxwell distribution. Molecular visualization was performed using Visual Molecular Dynamics software (41).

## RESULTS AND DISCUSSION

**Figure 1.**
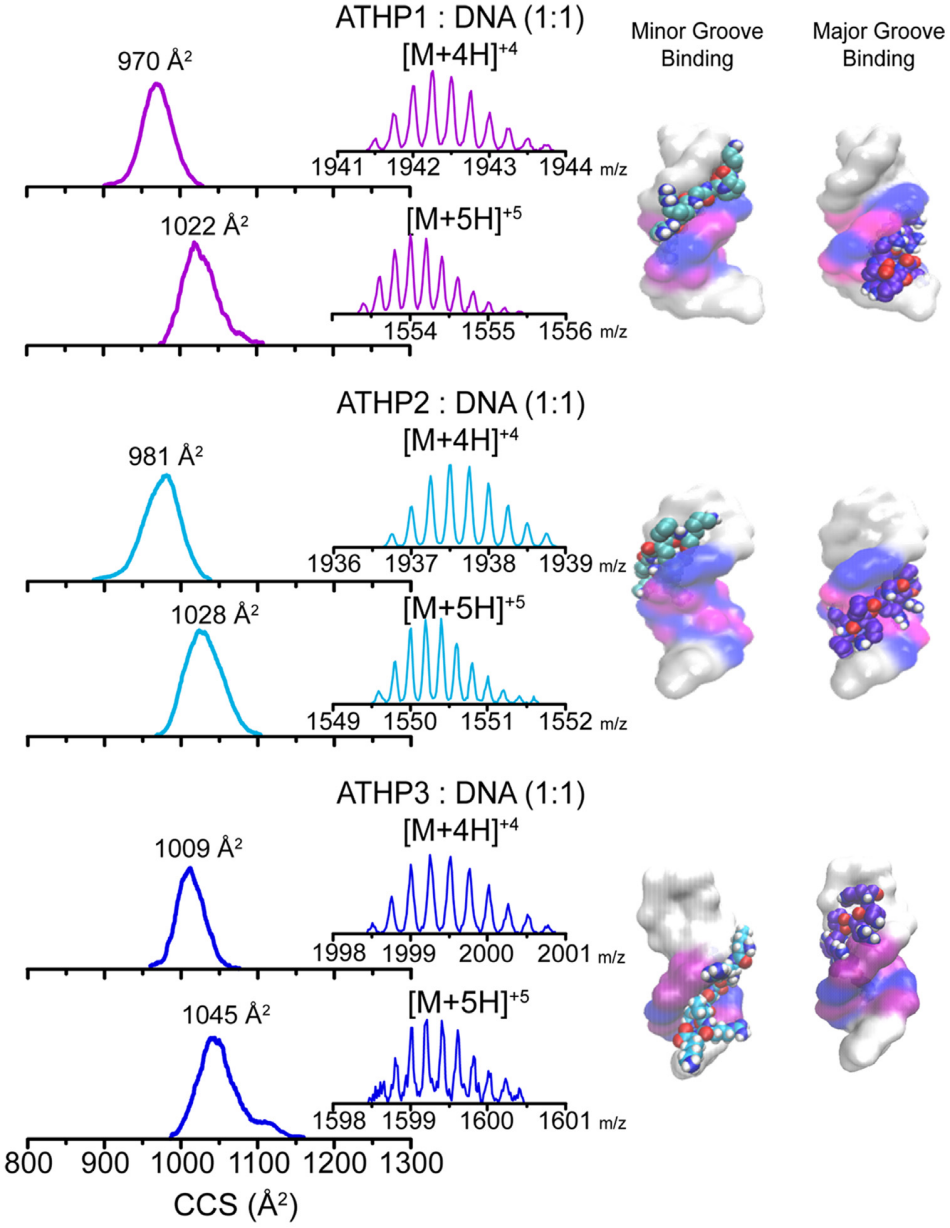
Typical mobility profiles, MS distributions (inset) and candidate structures (right) are shown for the [M + 4H]^+4^ and [M + 5H]^+5^ charge states of ATHP 1, 2, or 3 in complex with the DNA hairpin (FL876) with a ATHP: DNA 1:1 stoichiometry.

The native IMS-MS analysis of the ATHP 1, 2 or 3 in complex with the AT-rich DNA hairpin substrate (FL876 oligonucleotide that forms a stem-loop or hairpin structure and contains a single AT site for ATHP binding) produced [M + 4H]^+4^ and [M + 5H]^+5^ ions (Figure 1). The native IMS-MS analysis of equal molar sample ratios shows a complex formation with 1:1 ATHP peptide:DNA stoichiometry, accompanied by each species in their free form for all three DNA duplexes (Supplementary Figure S1-S3). Differences in the ATHPs:DNA duplexes binding were not observed depending on the hairpin sequence. Inspection of the mobility profiles of the ATHP:DNA complex shows a single mobility band for each charge state; this observation is indicative of a locked complex structure when compared to the several mobility bands observed for the ATHPs in the free form. Candidate structures based on molecular modeling were proposed based off these initial findings and suggest the likelihood of the ATHPs attaching to either the major or minor grooves of the DNA hairpin. The small difference in the CCS values of the candidate structures corresponding to the major or minor grooves ATHP:DNA 1:1 stoichiometry does not allows for their unambiguous assignment (Supplementary table S1); that is, a good agreement is observed for both candidate structures with the experimental mobility profiles. This contrasts previous assumptions in the literature (e.g., ATHPs are specific minor groove binding molecules) and subsequently motivated a series of experiments to evaluate the binding specificity of each motif.

**Figure 2.**
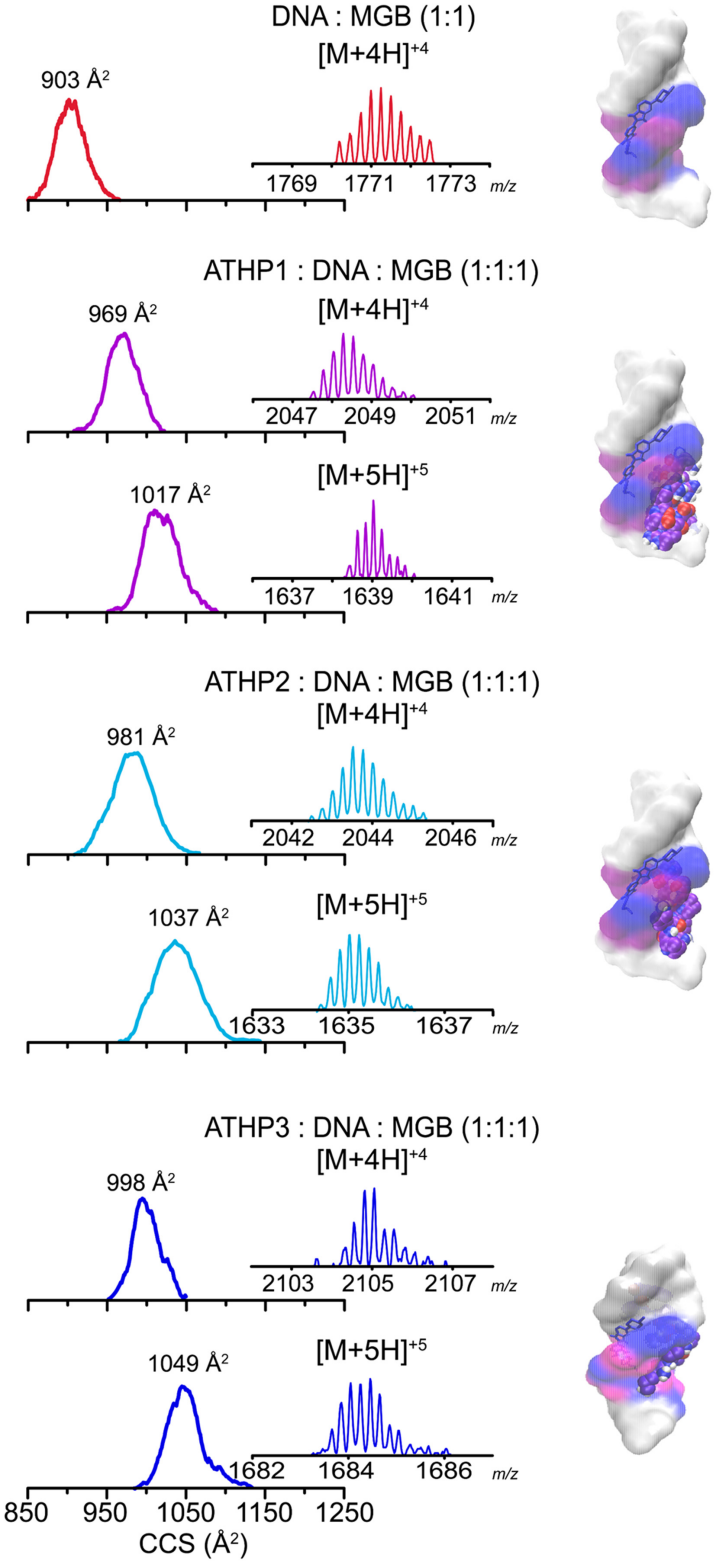
Typical mobility profiles, MS distributions (inset) and candidate structures (right) are shown for the [M + 4H]^+1^ and [M + 5H]^+5^ charge states of ATHP 1, 2, or 3 in complex with the DNA hairpin (FL876) and the minor groove binding (MGB) Hoechst 33258 with a ATHP: DNA : MGB 1:1:1 stoichiometry

We proceeded by using Hoechst 33528, a fluorescent dye known as a minor groove binding (MGB) compound that is highly specific to AT-rich DNA substrates and tightly binds to AT-rich DNA regions with a binding affinity in the range of 10^8^ M^–1^ (42). By blocking the minor groove, ATHP attachment was restricted to the major groove of FL876 so that the presence or absence of binding could be determined. Although displacement of the Hoechst 33258 compound in the minor groove by ATHPs is possible, we hypothesized that the majority of ATHPs would attach to the major groove, if applicable, leaving the minor groove occupied by the dye. In fact, native nESI-IMS-MS results confirmed that all three ATHPs have the ability to bind to the DNA (FL876): Hoechst complex, forming 1 : 1: 1 molecular assemblies (Figure 2). Inspection of the mobility profiles showed a single mobility band, also indicative of a locked 1:1:1 complex structure. The molecular modeling including the MGB compound supported the hypothesis of the additional ATHP major groove binding capabilities; a good agreement was observed between the theoretical CCS values from the candidate structures of the 1:1:1 DNA(FL876): Hoechst: ATHP complexes and the experimental mobility measurements (Supplementary Table S2).

**Figure 3.**
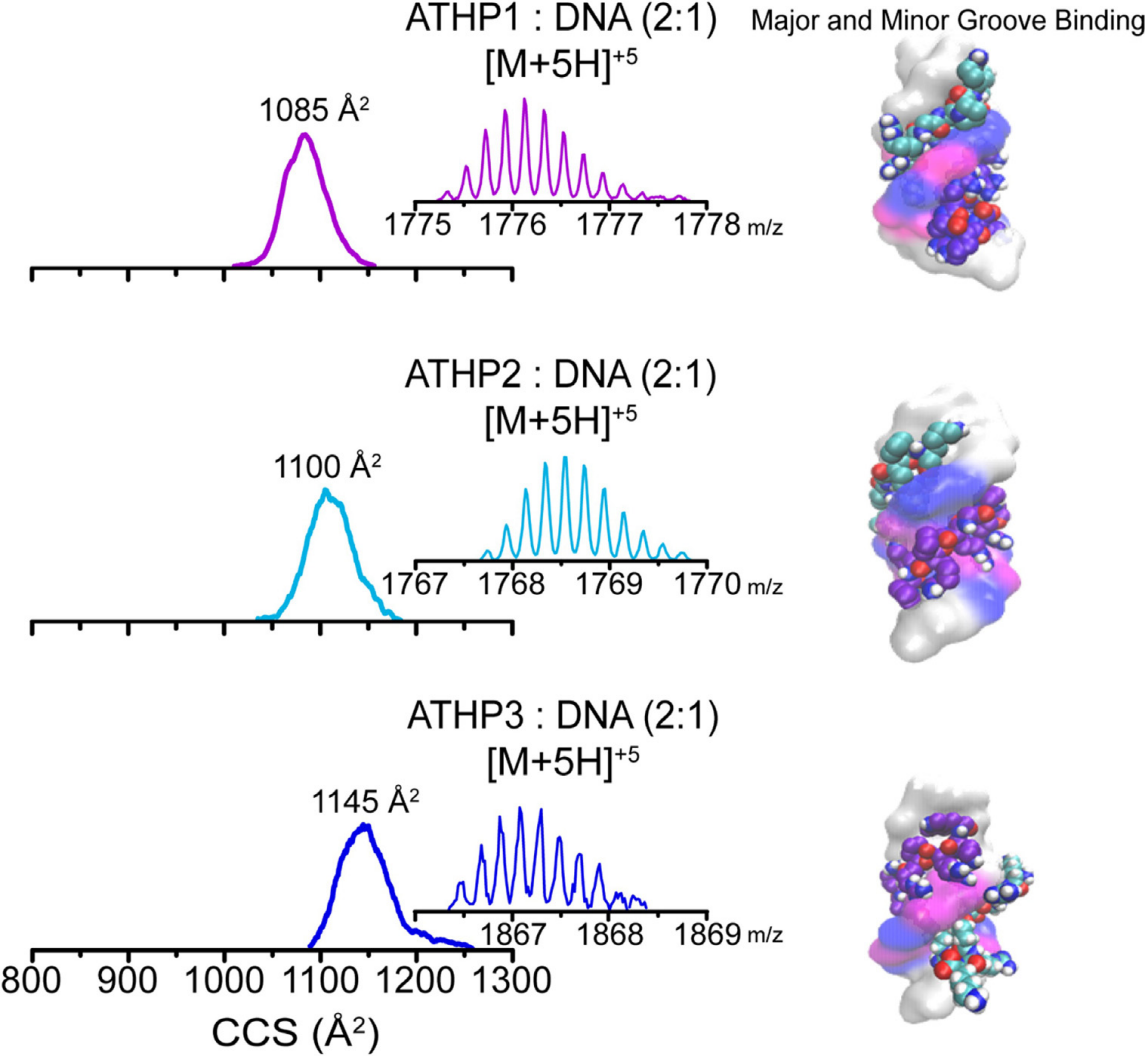
Typical mobility profiles, MS distributions (inset) and candidate structures (right) are shown for the [M + 4H]^+1^ and [M + 5H]^+5^ charge states of ATHP 1, 2, or 3 in complex with the DNA hairpin (FL876) with a ATHP: DNA 2:1 stoichiometry.

Upon confirmation of the interaction between ATHP and the major groove of FL876 DNA duplex, the peptide concentration was increased two-fold, with respect to DNA, to assess the possibility of two peptides with one DNA (bound to the major and minor grooves). An increase in the ATHP: DNA ration to 2:1 resulted in the observation of the corresponding [M + 5H]^+5^ complex (Figure 3 and Supplementary Figures S1-S3). The small difference between the CCS values (<200 Å^2^) between the ATHP: DNA 1:1 and 2:1 complexes suggest that the ATHP peptide(s) are mostly concealed within the DNA duplex structure. This is a common trend for the three considered DNA duplexes (Supplementary Figure S2 and S3) which suggest that there is no influence of the hairpin constituents in the ATHP-DNA binding dynamics. The proposed candidate structures provide an illustration of the ATHP:DNA (FL876) 2:1 complex; inspection of the candidate structures suggests the CCS is mostly dictated by the DNA substrate. A good agreement is also observed between the experimental CCS and those derived from the candidate structure 2:1 ATHP-DNA complexes (Supplementary Table S1). The IMS-MS results and theoretical docking studies are also in good agreement with previous structural findings using NMR and X-ray crystallography; that is, the –Arg-Gly-Arg– core of the AT-hook peptide can be buried into the minor groove of DNA, and that complexes are formed via hydrogen bonds between the oxygen atoms of thymine and/or cytosine, while Van der Waals forces govern interactions with adenines (24,26,27). The lysines and arginines outside of this core extend tightly along the DNA forming additional points of contact as a result of electrostatic interactions with the negatively charged DNA phosphate backbone (24,26,27).

**Scheme 2.**
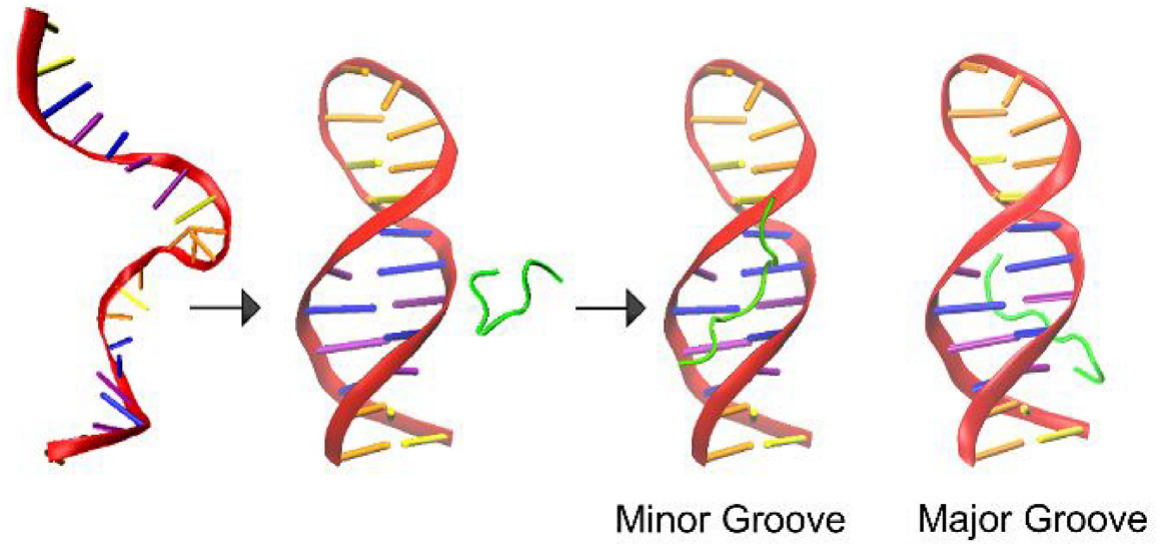
Mechanism of ATHP attachment to DNA showing the preformed hairpin prior to peptide unfolding upon binding.

Comparison of the mobility distributions of the 1:1 and 2:1 ATHP: DNA stoichiometries suggest that the ATHPs bind to the AT-rich regions of the DNA in a single ‘locked’ conformation and that all three ATHPs are selectively stabilized upon binding to the pre-formed FL876 hairpin (Scheme 2) (43). The schematic model, determined using the CCSs measured in the native IMS-MS, illustrates conformational changes of the peptide (i.e., unfolding) induced upon minor and/or major groove substrate binding (44). These observations suggest a disordered-to-ordered transition of HMGA2 when assemblies with DNA, where the binding occurs indistinctly via the minor or major groove.

The ATHP: DNA complexes were also studied using fluorescence titration measurements, DNA melting experiments, ITC, and collision induced dissociation MS/MS (MS-CID-MS). Supplementary Figure S4 shows results of fluorescence titration experiments where increasing concentrations of ATHP1, 2, and 3 were titrated into a Hoechst 33258-FL876 complex. Since Hoechst 33258 tightly binds to the minor groove of AT-rich DNA (42,45), ATHPs should not completely displace Hoechst 33258 from the minor groove. In this way, the fluorescence intensity of the Hoechst 33258-FL876 complex should not greatly decrease. Indeed, high concentrations of ATHPs (20 μM) could not fully quench the fluorescence of the Hoechst 33258-FL876 complex, indicating that ATHPs cannot completely remove Hoechst 33258 from the minor groove. This result is consistent with our previous IMS-MS studies.

**Figure 4.**
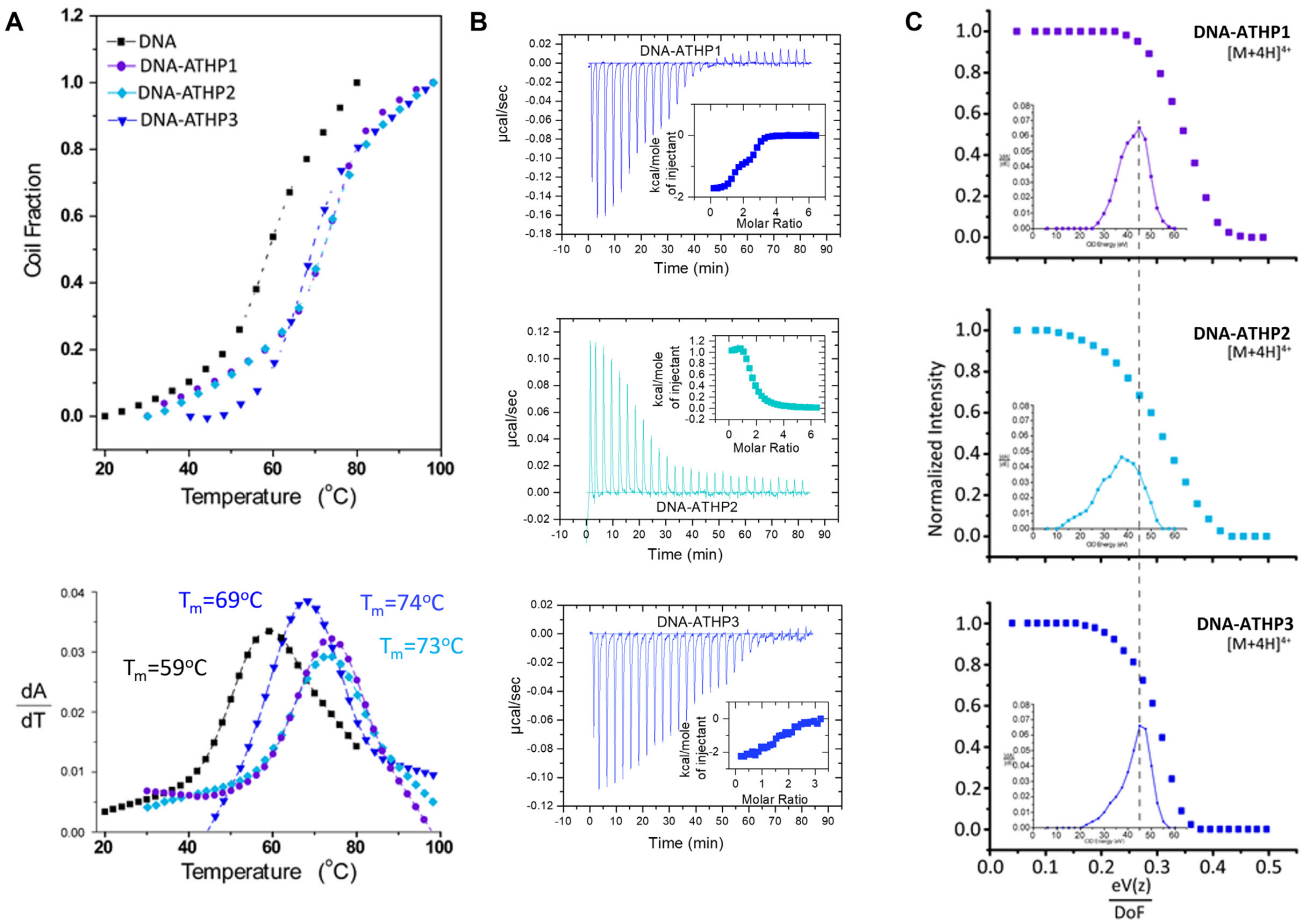
DNA UV melting curves for DNA hairpin (FL876) and DNA hairpin (FL876) complexed with ATHPs 1, 2 or 3 (**A**). ITC results determining the binding stoichiometry and affinity of ATHP 1, 2 and 3 to both the major and minor groove of FL876 DNA (**B**) and Collision induced dissociation for FL876 complexed with ATHP1. ATHP2 or ATHP 3 (charge state and degrees of freedom were considered) with the curve derivative shown as an inset (**C**).

Hairpin formation was confirmed by a sharp DNA UV melting transition at 59°C in 1 × BPE (see Figure 4A). The ATHPs 1- 3 showed a melting temperature (T_m_) increase, indicative of binding between each peptide and hairpin. The thermal stabilization of FL876 by ATHP 1 and ATHP 2 is greater than that caused by ATHP 3, indicating that ATHP1 and 2 may bind more tightly to FL876 than ATHP3 does.

The ITC results also supported the dual binding site hypothesis. Figure 4B shows the ITC results in which all can be fitted to a two-site model. Binding of ATHP1 to FL876 results in n1 of 1.3 ± 0.1, K1 of 1.9 ± 1.6 × 10^8^ M^–1^, ΔH1 of -1.7 ± 0.7 kcal/mol, n2 of 1.4 ± 0.1, K2 of 4.0 ± 2.4 × 10^6^ M^–1^, and ΔH1 of -0.9 ± 0.1 kcal/mol; binding of ATHP2 to FL876 results in n1 of 0.8 ± 0.5, K1 of 3.9 ± 2.4 × 10^5^ M^–1^, ΔH1 of + 1.6 ± 0.7 kcal/mol, n2 of 0.7 ± 0.3, K2 of 9.4 ± 4.1 × 10^6^ M^–1^, and ΔH1 of + 1.0 ± 0.1 kcal/mol; and binding of ATHP3 to FL876 results in n1 of 1.3 ± 0.4, K1 of 1.1 ± 1.3 × 10^7^ M^–1^, ΔH1 of -2.4 ± 0.2 kcal/mol, n2 of 1.1 ± 0.2, K2 of 1.1 ± 1.0 × 10^6^ M^–1^, and ΔH1 of -1.1 ± 0.7 kcal/mol. The binding enthalpy values are quite small, which gives significant uncertainty to the estimate of the binding affinities. According to our previous studies, the binding affinity of ATHPs to AT-rich DNA sequences should be in the range of 10^6^ to 10^7^ M^–1^ (46). Interestingly, binding of ATHP1 and 3 to FL876 is an exothermic reaction. In contrast, binding of ATHP2 to FL876 is an endothermic reaction. These results are consistent with our previously published results (46,47).

Complementary collision induced activation experiments (MS-CID-MS) on the [M + 4H]^+4^ charge state of ATHP and FL876 1:1 complexes, were used to evaluate the ATHP-DNA binding affinities (Figure 4C). As the collision energy increases, a decrease in the molecular ion signal corresponding to the complex was observed; notice that collision energy has been normalize to the charge and calculated per degree of freedom to account for mass difference and charge between the experiments, a consideration supported through previous work by Clemmer's and co-workers (48). Comparison between the CID profiles of the ATHP 1–3: FL876 complexes shows that the weakest binding profile is observed for ATHP 2: FL876, followed by ATHP 3: FL876 complex, and ATHP 1: FL876 complex. We interpret these CID profiles in that as the collision energy increases, multiple collisions with the collision gas (nitrogen in this case) leads to an increase in the vibrational modes of the complex eventually leading to dissociation into the main constituents (i.e., ATHP and FL876). That is, as the CID energy increases the internal energy increases until a threshold is achieved, leading to complex dissociation. The increased energy needed to begin dissociation of ATHP 1: FL876 and ATHP 3: FL876 distribution suggests that two distinct binding sites (e.g., minor and major groove) are occupied. The more immediate dissociation of the ATHP 2-FL876 complex suggest a predominant single binding site or two weaker attachment sites. The CID profiles combined with the fluorescent titration and ITC data indicate that ATHP 1, 2 and 3 can bind hairpin DNA and occupy both minor and major grooves.

## CONCLUSIONS

These studies evaluated the binding dynamics of ATHP 1, 2 or 3 of HMAG2 in complex with an AT-rich DNA hairpin duplex (FL876). Results showed that ATHP 1–3 can associate with the AT-rich DNA to form a complex in a ‘locked’ mechanism enabled by interactions of the –Arg-Gly-Arg– tripeptide core and the AT-rich regions of the DNA duplex. The use of DNA duplexes with varying hairpin constituents showed no differences in the ATHPs :DNA complex formation. The native IMS-MS, combined with the fluorescent titration and ITC data indicate that ATHP 1, 2 and 3 can bind to DNA duplexes and occupy both minor and major grooves in a 1:1 and 2:1 ATHP: DNA stoichiometry. Molecular modeling supported the observation of the ‘locked’ complex structure, where the ATHPs are likely to bind to a pre-folded DNA duplex structure in the minor and major grooves, indistinctively. Complementary ATHP 1–3: FL876 complex analysis using MS-CID-MS showed that the weakest binding profile is observed for ATHP 2 : FL876, followed by ATHP 3 : FL876 complex, and ATHP 1: FL876 complex.

## Supplementary Material

gkac115_Supplemental_Files
