# Supplementary material for: AT-hook peptides bind the major and minor groove of AT-rich DNA duplexes
Source: Nucleic Acids Res. 2022 Feb 25;50(5):2431–9. doi: 10.1093/nar/gkac115 (PMC8934665; doi:10.1093/nar/gkac115)
Supplement: gkac115_Supplemental_Files [file gkac115_supplemental_files.zip › Supplementary Data-Figures_Final.pdf]

AT-hook peptides bind the major and minor groove of AT-rich DNA duplexes

Alyssa Garabedian[1], Kevin Jeanne Dit Fouque[1,3], Prem P. Chapagain[2,3], Fenfei Leng[1,3], and Francisco Fernandez-Lima[1,3]*.

[1] Department of Chemistry and Biochemistry, Florida International University, Miami, 33199 USA

[2] Department of Physics, Florida International University, Miami, 33199 USA

[3] Biomolecular Sciences Institute, Florida International University, Miami, 33199 USA

* To whom correspondence should be addressed. Tel: 305-348-2037 Email: fernandf@fiu.edu

SUPPLEMENTARY DATA

Table of contents:

Scheme S1. DNA duplexes utilized in this study FL876, FL876T1 and FL876T2

Figure S1. Typical mass spectra of 1:1 and 2:1 ATPH:DNA (FL876) complex

Figure S2. Typical mass spectra (A) and mobility profiles(B) of 1:1 and 2:1 ATPH:DNA (FL876T1) complex

Figure S3. Typical mass spectra (A) and mobility profiles (B) of 1:1 and 2:1 ATPH:DNA (FL876T2) complex

Figure S4. Fluorescent measurements for DNA (FL876) and Hoechst 33258 dye titrated with ATHP 1, ATHP 2 or ATHP 3.

Table S1. Experimental and theoretical mobility and CCS values for the ATHPs 1, 2 and 3 in complex with the DNA hairpin (FL876) and the Hoechst 33258.

Table S2. Experimental and theoretical mobility and CCS values for the ATHPs 1, 2 and 3 in complex with the DNA hairpin (FL876T1 and FL876T2).

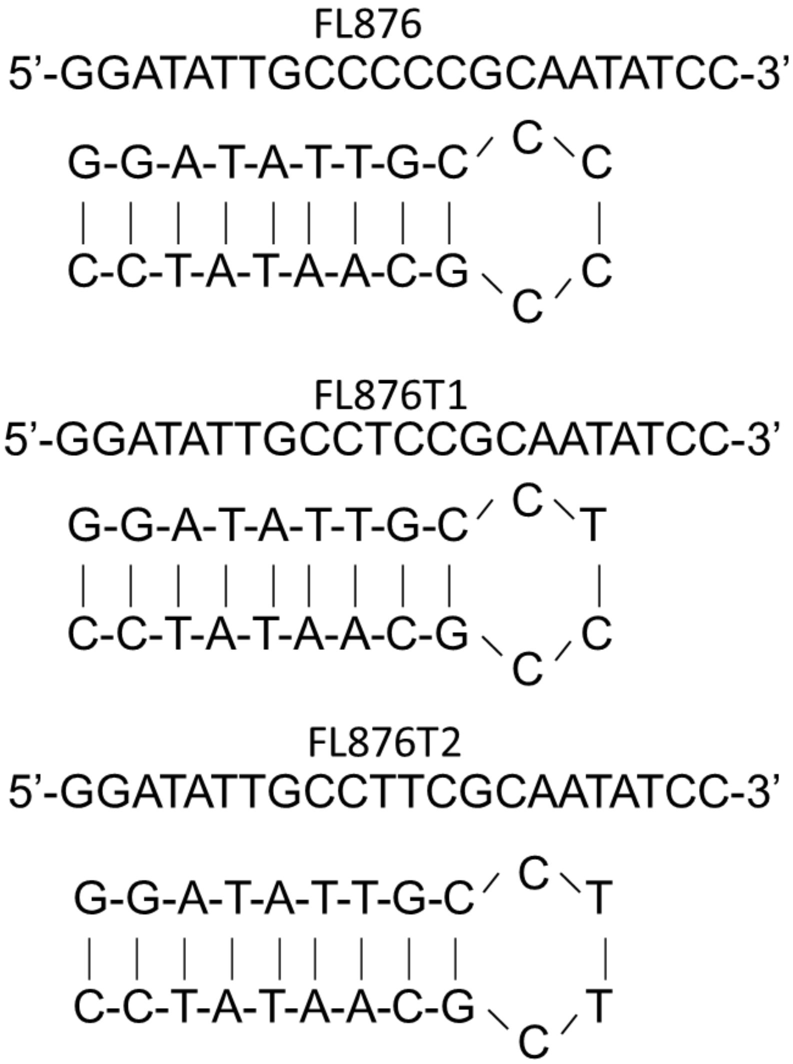

Scheme S1. DNA duplexes utilized in this study FL876, FL876T1 and FL876T2

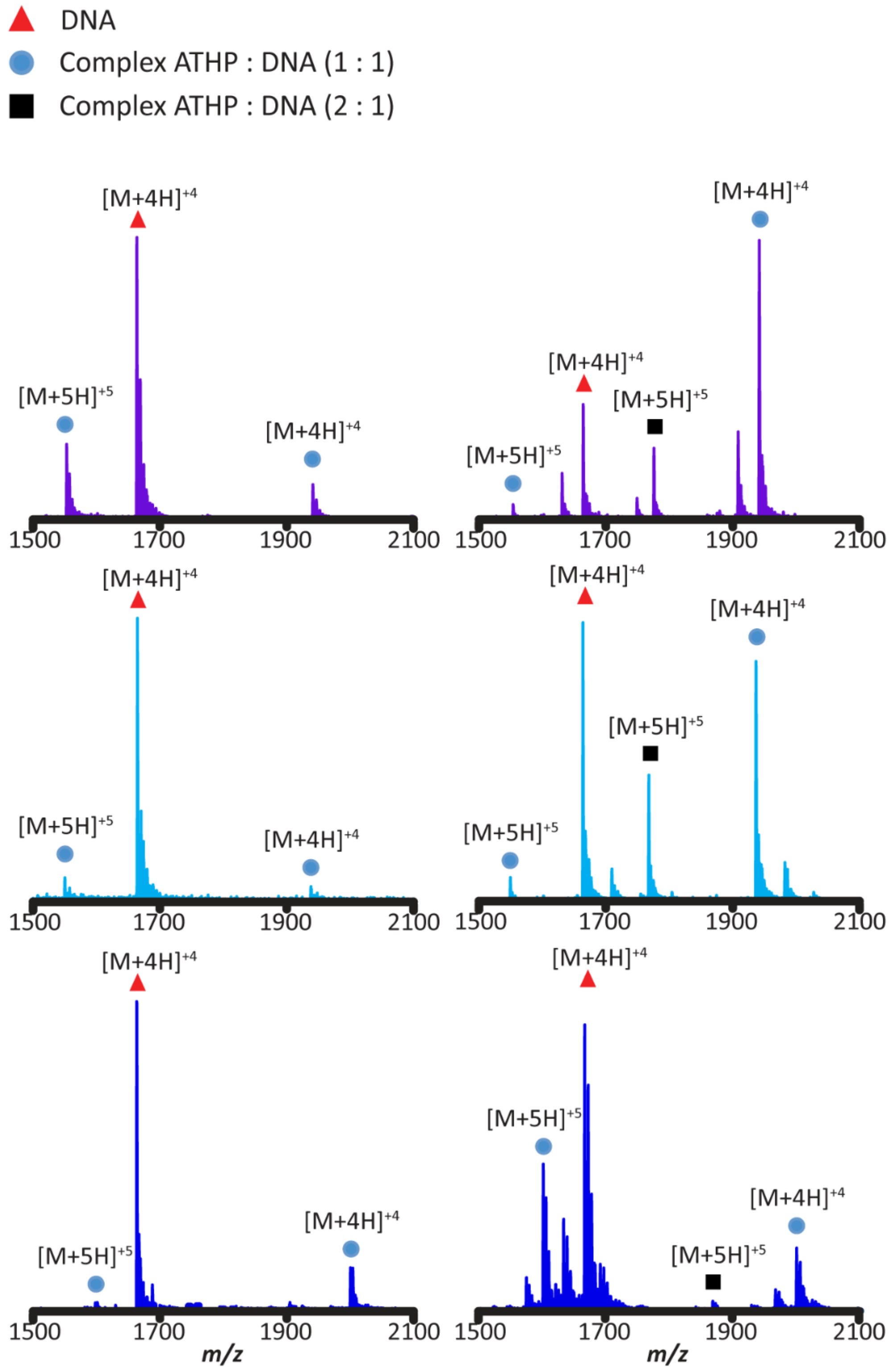

Figure S1. Typical mass spectra of 1:1 and 2:1 ATPH:DNA (FL876) complex.

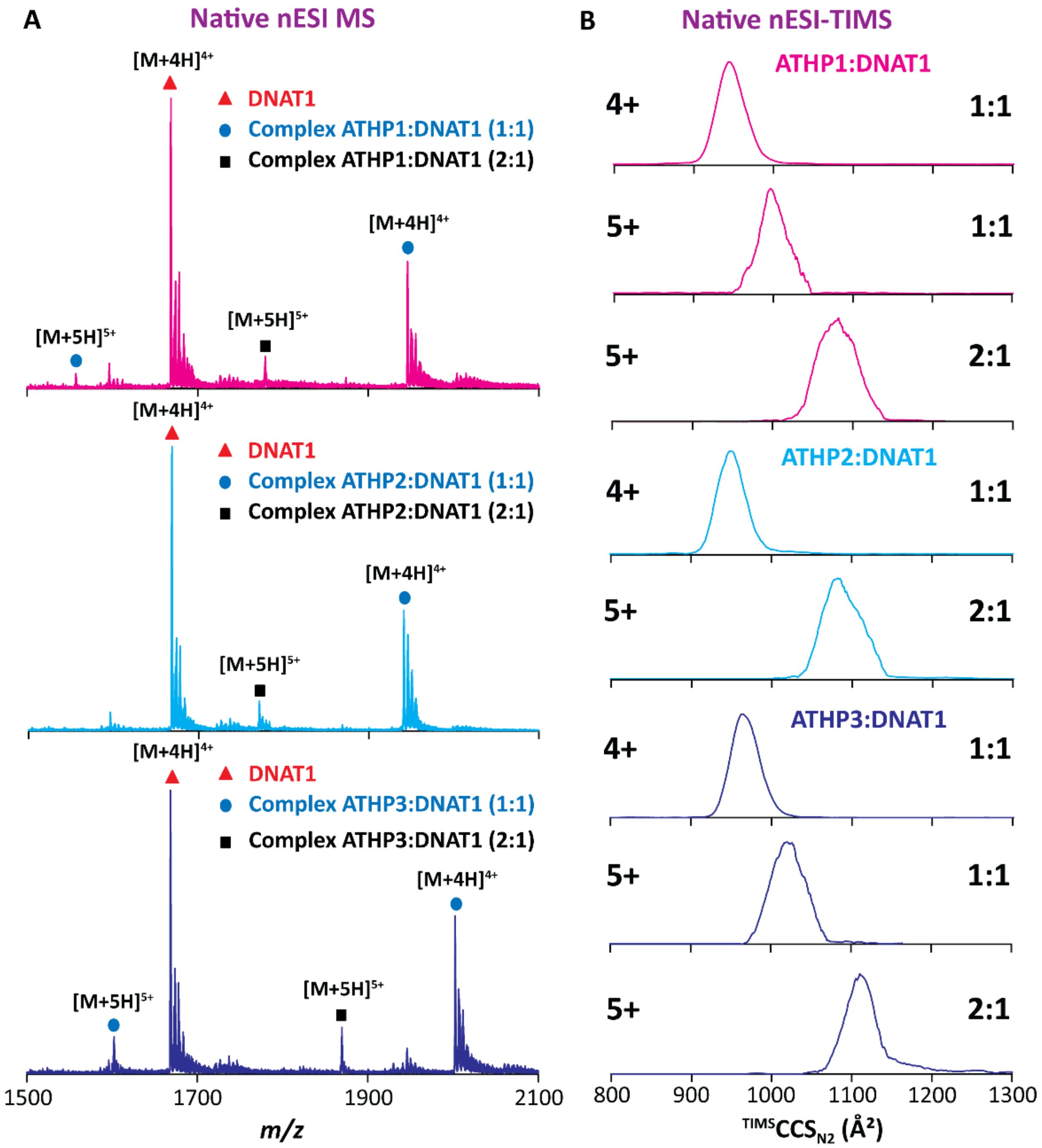

Figure S2. Typical mass spectra (A) and mobility profiles(B) of 1:1 and 2:1 ATPH:DNA (FL876T1) complex

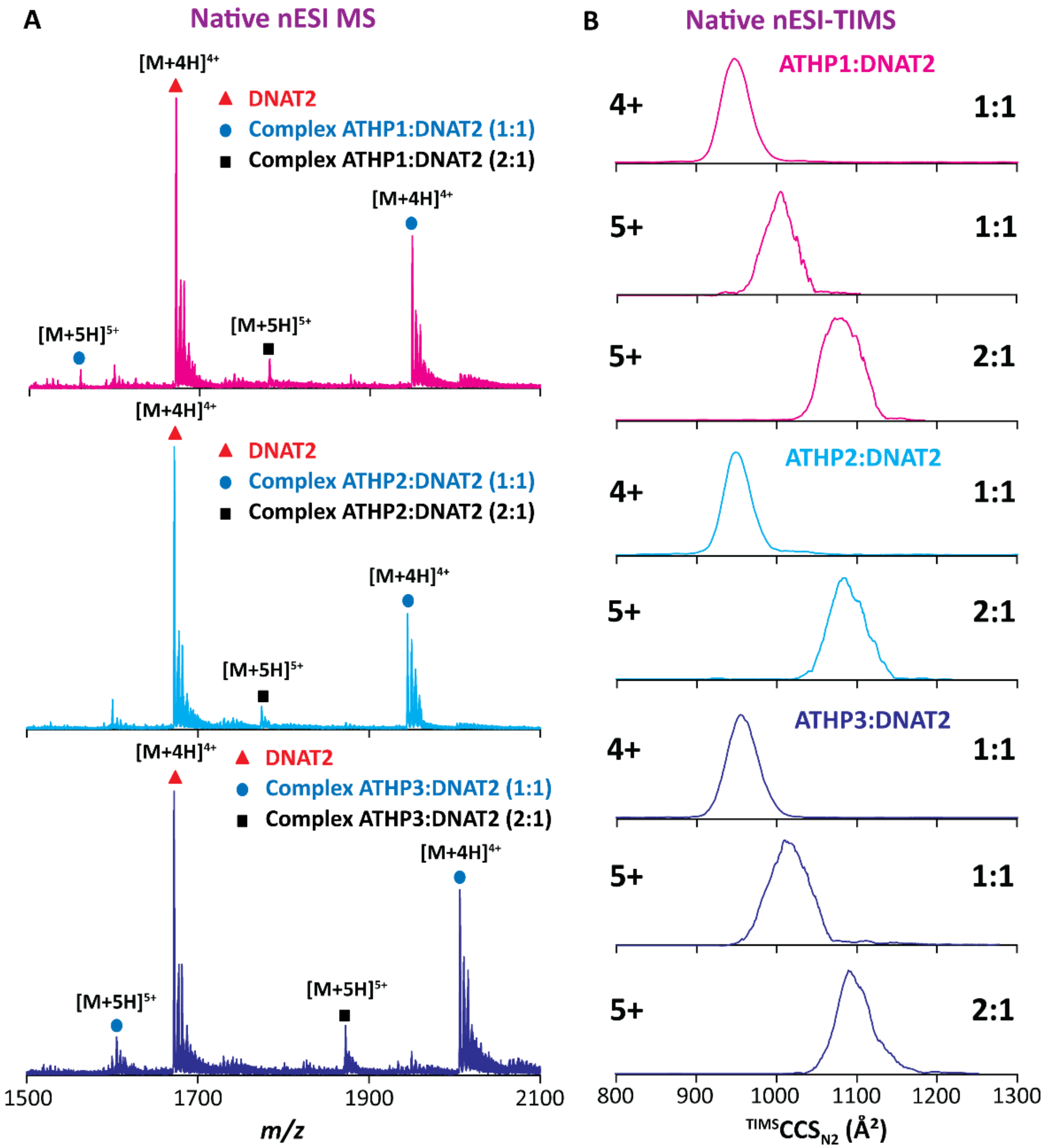

Figure S3. Typical mass spectra(A) and mobility profiles(B) of 1:1 and 2:1 ATPH:DNA (FL876T2) complex

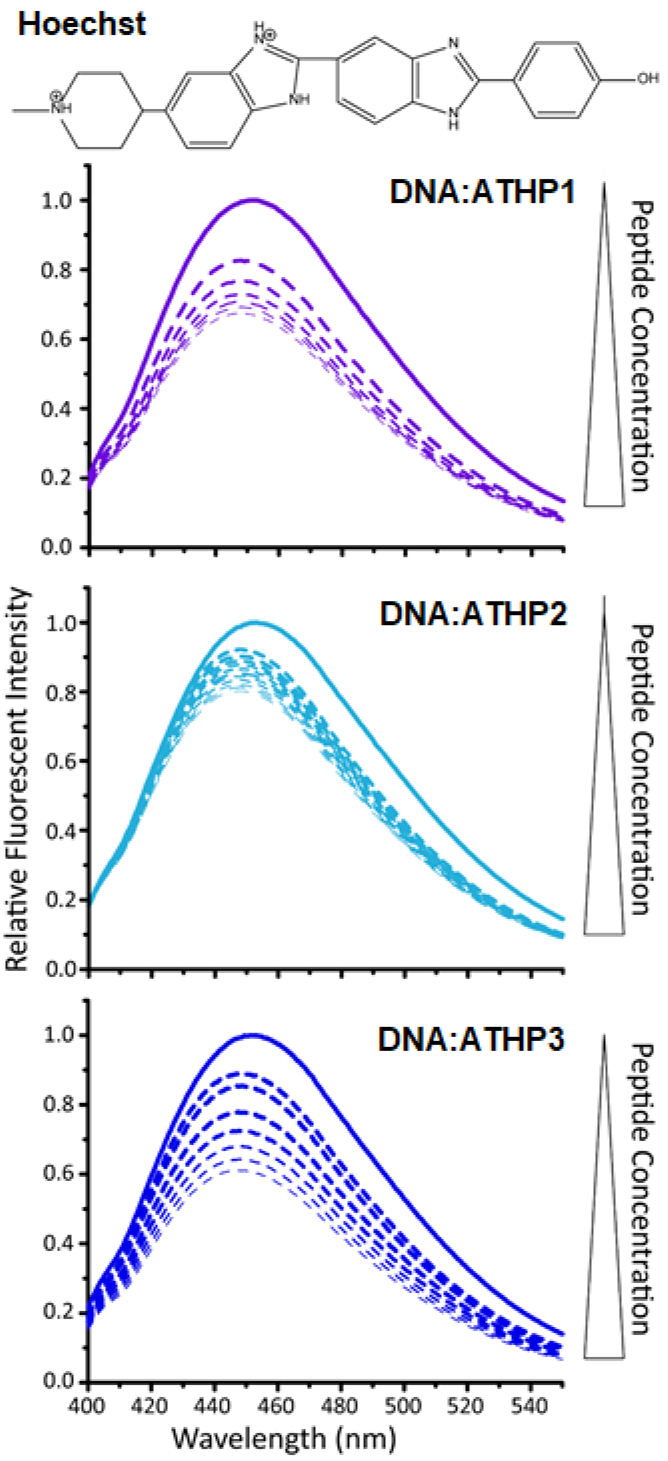

Figure S4. Fluorescent measurements for DNA (FL876, 100 nM) and Hoechst 33258 dye (50 nM) titrated with ATHP 1, ATHP 2 or ATHP 3 to 20 μM in 1×BPE.

Table S1. Experimental and theoretical mobility and CCS values for the ATHPs 1, 2 and 3 in complex with the DNA duplex (FL876) and the Hoechst 33258.

**FL876-AT-Hook1 complex $[M+nH]^{+n}$**		**TIMS Experimental**		**Theoretical (Major/minor)**
z	ratio	K_0 (cm^2 V^{-1} s^{-1})	CCS_{N2} ($Å^2$)	CCS_{N2} ($Å^2$)
4	1:1	0.828	970	1003/1047
5	1:1	0.982	1022	1043/1086
5	1:2	0.925	1085	1131
**FL876-AT-Hook2 complex $[M+nH]^{+n}$**				
4	1:1	0.819	981	1007/1045
5	1:1	0.977	1028	1045/1083
5	1:2	0.905	1110	1154
**FL876-AT-Hook3 complex $[M+nH]^{+n}$**				
4	1:1	0.796	1009	1041/1049
5	1:1	0.961	1045	1078/1088
	1:1	0.905	1110	
	1:1	0.827	1214	
5	1:2	0.877	1145	1196
**FL876-AT-Hook1-Hoechst 33258 complex $[M+nH]^{+n}$**				
4	1:1	0.829	969	1001
5	1:1	0.987	1017	1044
**FL876-AT-Hook2-Hoechst 33258 complex $[M+nH]^{+n}$**				
4	1:1	0.819	981	1110
5	1:1	0.959	1037	1044
**FL876-AT-Hook3-Hoechst 33258 complex $[M+nH]^{+n}$**				
4	1:1	0.805	998	1008
5	1:1	0.957	1049	1045
**FL876-Hoechst 33258 complex $[M+nH]^{+n}$**				
4	1:1	0.894	903	938

Table S2. Experimental and theoretical mobility and CCS values for the ATHPs 1, 2 and 3 in complex with the DNA hairpin (FL876T1 and FL876T2).

**FL876T1-AT-Hook1 complex $[M+nH]^{+n}$**		**TIMS Experimental**	
z	ratio	K_0 ($cm^2 V^{-1} s^{-1}$)	CCS_{N2} ($Å^2$)
4	1:1	0.851	946
5	1:1	1.008	998
5	1:2	0.925	1085
**FL876T1-AT-Hook2 complex $[M+nH]^{+n}$**			
4	1:1	0.842	953
5	1:2	0.921	1093
**FL876T1-AT-Hook3 complex $[M+nH]^{+n}$**			
4	1:1	0.834	965
5	1:1	0.981	1025
5	1:2	0.911	1116
**FL876T2-AT-Hook1 complex $[M+nH]^{+n}$**			
4	1:1	0.848	948
5	1:1	1.001	1006
5	1:2	0.931	1080
**FL876T2-AT-Hook2 complex $[M+nH]^{+n}$**			
4	1:1	0.847	948
5	1:2	0.923	1087
**FL876T2-AT-Hook3 complex $[M+nH]^{+n}$**			
4	1:1	0.842	956
5	1:1	0.977	1017
5	1:2	0.917	1093
